# Reaction Model Taking into Account the Catalyst Morphology and Its Active Specific Surface in the Process of Catalytic Ammonia Decomposition

**DOI:** 10.3390/ma14237229

**Published:** 2021-11-26

**Authors:** Walerian Arabczyk, Rafał Pelka, Izabella Jasińska, Zofia Lendzion-Bieluń

**Affiliations:** 1 Department of Inorganic Chemical Technology and Environment Engineering, Faculty of Chemical Technology and Engineering, West Pomeranian University of Technology in Szczecin, Piastów Ave. 42, 71-065 Szczecin, Poland; walerian.arabczyk@zut.edu.pl (W.A.); Zofia.Lendzion-Bielun@zut.edu.pl (Z.L.-B.); 2 Grupa Azoty Zakłady Chemiczne “Police” S.A., 1 Kuźnicka Str., 72-010 Police, Poland; izabella.jasinska@grupaazoty.com

**Keywords:** iron catalyst, nitriding process, kinetics, morphology, ammonia

## Abstract

Iron catalysts for ammonia synthesis/nanocrystalline iron promoted with oxides of potassium, aluminum and calcium were characterized by studying the nitriding process with ammonia in kinetic area of the reaction at temperature of 475 °C. Using the equations proposed by Crank, it was found that the process rate is limited by diffusion through the interface, and the estimated value of the nitrogen diffusion coefficient through the boundary layer is 0.1 nm^2^/s. The reaction rate can be described by Fick’s first equation. It was confirmed that nanocrystallites undergo a phase transformation in their entire volume after reaching the critical concentration, depending on the active specific surface of the nanocrystallite. Nanocrystallites transform from the α-Fe(N) phase to γ’-Fe_4_N when the total chemical potential of nitrogen compensates for the transformation potential of the iron crystal lattice from α to γ; thus, the nanocrystallites are transformed from the smallest to the largest in reverse order to their active specific surface area. Based on the results of measurements of the nitriding rate obtained for the samples after overheating in hydrogen in the temperature range of 500–700 °C, the probabilities of the density of distributions of the specific active surfaces of iron nanocrystallites of the tested samples were determined. The determined distributions are bimodal and can be described by the sum of two Gaussian distribution functions, where the largest nanocrystallite does not change in the overheating process, and the size of the smallest nanocrystallites increases with increasing recrystallization temperature. Parallel to the nitriding reaction, catalytic decomposition of ammonia takes place in direct proportion to the active surface of the iron nanocrystallite. Based on the ratio of the active iron surface to the specific surface, the degree of coverage of the catalyst surface with the promoters was determined.

## 1. Introduction

Nanomaterials have been intensively studied in recent years in the field of nanotechnology, catalysis, medicine, and others [1,2,3,4]. Precise determination of particle or grain size distribution (GSD) is of great importance to many industries, especially when dealing with nanomaterials. It is related to the fact that physical (e.g., magnetic characteristics, melting temperature, and absorption of electromagnetic waves) and chemical (obtaining of new materials, activity, and selectivity of catalysts) properties of such materials are a consequence of the size of the nanoparticles [3,5,6,7,8,9,10,11,12]. If so, providing only the mean value of the nanocrystallite sizes is not sufficient to define the full characteristics of the substance studied. This is crucial if we want to understand better surface phenomena occurring on nanoparticles under process conditions, particularly in heterogeneous catalysis [13,14,15,16,17,18,19,20,21,22,23,24,25,26,27,28,29]. Catalyst testing techniques often deviate from the conditions under which the catalytic processes are run. Therefore, in situ methods of examining catalysts are especially useful.

Processes taking place on the iron catalyst have been studied extensively (nanoFe-NH_3_-H_2_ system) [13,14,15,16]. Catalyst for the synthesis of ammonia, which, in addition to iron, contains promoters, was tested in terms of understanding the mechanism of synthesis and decomposition of ammonia [13,14,30,31], as well as with the initial stage of the nitriding process [32,33,34]. As a result of these studies, it was found that measuring the nanocrystallite size distribution is essential for proper examination of a Fe-NH_3_-H_2_ system because of its nanocrystalline structure. 

Based on the kinetic studies of the nitriding process of the iron catalyst, i.e., the system of parallel reactions—nitriding and catalytic decomposition of ammonia [33,35]—, a model of the reaction between the nanocrystalline solid phase and the gas phase was developed—a model of the reaction in the adsorption range [34,36]. It was found that nanocrystallites of α-Fe transform to γ’-Fe_4_N nitride phase in all their volume, in order, according to their size, from the smallest to the largest [34,36].

Based on the above research, two methods (chemical ones) were developed to measure the iron nanocrystallites size distribution.The first way is to measure (by XRD) the average nanocrystallite size (of the substrate or the product) [37]. The second option uses the measurement of the chemical reaction rate corresponding to given conversion degrees, α [38].

It was determined [32,39,40,41,42] that during nitriding processes of iron catalyst for ammonia synthesis (using hydrogen-ammonia gaseous mixtures with increasing nitriding potentials; temperature is constant) stationary states were established at a given nitriding potential, P_0_. In these stationary states, the parallel reaction of catalytic decomposition of ammonia proceeds at a constant rate, and the nitriding reaction rate equals zero at P(t = ∞) = P_0_. 

The P_0_ potential depends on the size of nanocrystallites [41,43]. Despite the multiple repetition of the nitriding α → γ’ and reduction γ’ → α cycles at high temperatures, the structure of the catalyst does not change. As the nitriding potential increases or decreases, nanocrystallites undergo a phase transformation, from the largest to the smallest [44,45]. In the nitriding reaction and reduction of nitrides in chemical equilibrium states, the phenomenon of hysteresis was demonstrated [39], with the nitriding potential being greater during the nitriding process than during the reduction process.

At a constant potential, the α-Fe(N) nanocrystallites can be in equilibrium with the γ’-Fe_4_N nanocrystallites, which cannot be explained on the basis of the Lehrer diagram [46]. The phenomenon was explained by taking into account an additional parameter in the Gibbs phase rule—the size of nanocrystallites [39,44,45].

Using the above findings, a third method (also chemical) for measuring the size distribution of nanocrystallites was invented [47]. This method is based on determining the change in the degree of reaction (corresponding to values of the gas phase chemical potential) in the stationary states. Relative size distribution is determined. When the size of the smallest or the largest nanocrystallite (using SEM, TEM, etc.) or the average crystallite size (XRD), or specific surface area (BET), are determined, it is then possible to determine the real nanoparticles size distribution of the sample tested.

The aim of the work was to develop a reaction model taking into account the morphology of catalysts and the degree of surface coverage with promoters, which could be used to describe the kinetics of ammonia decomposition. Based on this model, a new method for the characterization of iron catalysts allows for determination of the probability of nanocrystallite distribution density (PDF) according to their active surface area under the conditions of chemical reaction.

## 2. Experiment

A pre-reduced iron catalyst for ammonia synthesis was used in the experiments. Chemical composition of catalyst samples was determined by Inductively Coupled Plasma method (ICP-OES, spectrometer Perkin Elmer, type Optima 5300DV). It was found that the catalyst, apart from metallic iron, consisted of promoters in an amount of 3.3 wt.% Al_2_O_3_, 2.8 wt.% CaO, 0.7 wt.% K_2_O.

Process of reducing a passive layer of the catalyst, as well as heating the catalyst under reducing conditions and nitriding were carried out in a differential tubular reactor equipped with a system, enabling to conduct thermogravimetric measurements (with an accuracy of 1 × 10^−4^ g) and gas phase chemical composition analysis (hydrogen concentration in a gas phase was determined with an accuracy of 0.02 vol. %) [11]. Gas directed to the katharometric analyzer was taken from points in the immediate vicinity of the catalyst bed. Ammonia concentration in the gas phase was calculated based on a reactor mass balance. Reactant gas flow rates were determined by electronic mass flow controllers. Samples of ca 1 g with a grain size in the range of 1.0–1.2 mm were placed as a single layer of grains in a platinum basket hanging on the arm of a thermobalance. During the experiments, conditions for the process taking place in the kinetic region of the reaction are met.

During the reduction of a passive layer of the catalyst, the reactor was heated to a temperature of 500 °C at the rate of 10 °C min^−1^ with a hydrogen flow of 150 cm^3^ min^−1^. At the temperature of 500 °C, the weight of the catalyst stabilized and did not change, even after increasing the process temperature.

The reduced sample was nitrided at 475 °C with ammonia (200 cm^3^ min^−1^; 100% of ammonia at the reactor inlet) until the γ’-Fe_4_N nitride phase was obtained. The nitride was then reduced and the sample overheated at 550 °C, 600 °C, 650 °C, and 700 °C sequentially for ca. 17 h under constant flow of hydrogen. After each annealing at a specified temperature, the sample was nitrided at 475 °C. After the last nitriding, the sample was reduced at the process temperature, and the specific surface area was measured using BET method.

The aforementioned processes were carried out in an identical manner, periodically determining its specific surface area set at the aforementioned temperatures.

In order to check the structure stability, the samples were reduced and annealed at the temperature of 700 °C for 17 h, then tempered for 50 h at 475 °C, and the specific surface area was measured.

BET measurements were carried out on an automated AutoChem II 2920 apparatus, Micromeritrics, Norcross, GA, USA. Based on the measurements of the specific surface area, it was found that, after the structure was established at a given temperature, it was stable up to that temperature. The formation process of a structure at a given temperature is irreversible.

## 3. Results

**Figure 1:**
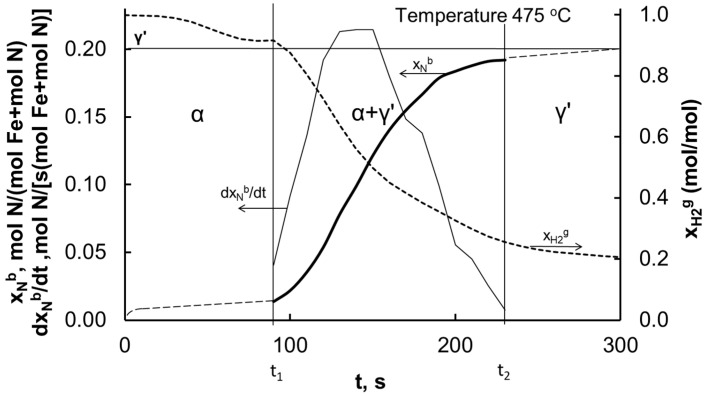
Nitriding degree, nitriding reaction rate, and hydrogen concentration as functions of time in nitriding process.

Figure 1 shows an example of the measurement of changes in nitrogen concentration in a solid phase and hydrogen concentration in a gas phase in the nitriding process of nanocrystalline iron with ammonia at 475 °C. The vertical lines indicate the reaction times of the smallest and largest iron nanocrystallites in the catalyst sample.

**Figure 2:**
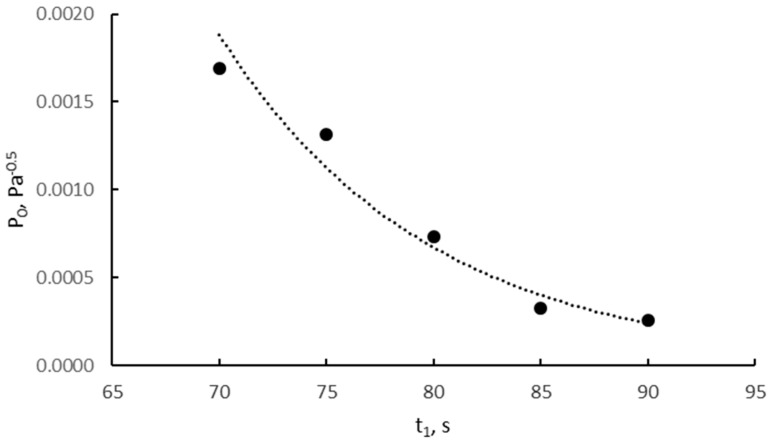
The minimum nitriding potential at which the nitriding of the smallest iron nanocrystallites begins in the samples reduced and heated in hydrogen in the temperature range of 500–700 °C.

Figure 2 shows the time after which, in the gas phase, the minimum nitriding potential, P_0_, is reached, at which the nitriding of the smallest iron nanocrystallites begins in the samples reduced and annealed in hydrogen in the temperature range of 500–700 °C.

## 4. Discussion

Phase transition of an individual α-Fe(N) nanocrystallite is an adiabatic, isobaric, and isosteric process, without nanocrystallite mass and energy exchange with the environment. With change in free enthalpy of phase transition, it is compensated by changes in free enthalpy related to nitrogen sorption in iron nanocrystallites and change in surface energy of iron nanocrystallite. At x_N,i_^s^ = A_i_ = S_a,i_/V_i_ [nm^−1^] (where A_i_—active specific surface of i-th single nanocrystallite [nm^−1^], S_a,i_—active surface area [nm^2^], V_i_—volume of a nanocrystallite [nm^3^]) on α-Fe(N) phase, there is a phase change α-Fe(N) → [γ’]^*^-Fe_4-x_N (where x—defects concentration). In the phase transformation, the segregation enthalpy changes, and the chemisorbed nitrogen dissolves in the volume of [γ’]^*^-Fe_4-x_N nanocrystallite and the surface concentration x_N,i_^s^ → 0.

Change in the deformation potential of the crystal lattice related to the critical concentration of nitrogen in iron of saturated α-Fe(N) with concentration x_N,i_^b,α,cri^ to the unsaturated phase [γ’]^*^-Fe_4-x_N indicates by how much the energy of a single nanocrystallite must increase, so that it can undergo the phase transition. The energy is supplied for the system with the increasing nitriding potential of a gas phase. Nanocrystallites with the largest size (the smallest A_i_) show the lowest energy barrier [44], so that they undergo the phase transition at lower nitriding potentials of gas phase. This indicates the direction of the nitriding reaction, which occurs in the order from the largest to the smallest crystallites (from the smallest to the largest values of A_i_) with gradually increasing nitriding potential of gas phase when P(t) ≈ P_0_(A_i_). For a given nitriding potential, the surface of all nanocrystallites is covered to the same extent. However, nanocrystallites will differ in volume and, therefore, nitrogen concentration in volume.

Total change in the Gibbs energy of the Fe-N system, ∆G, is zero:

∆G_Fe,i_^b,α-[γ]*^ + ∆G_N,i_^b^ + ∆G_N,i_^s^ + ∆G_Fe,i_^s^ = 0, (1)

where:

1. ∆G_Fe,i_^b,α-[γ]*^—change in free enthalpy of crystal lattice of phases α-Fe(N) → [γ’]^*^-Fe_4-x_N,
2. ∆G_N,i_^b^—change in free enthalpy of nitrogen dissolved in a volume of iron nanocrystallite,
3. ∆G_N,i_^s^—change in free enthalpy of nitrogen chemisorbed on α-Fe(N) nanocrystallite surface, and
4. ∆G_Fe,i_^s^—change in surface energy of iron nanocrystallite at the transformation, selecting chemical potentials of nitrogen and iron at a temperature 475 °C as reference conditions.

For a single i-th iron nanocrystallite, the equation describing the nitriding reaction rate can be written as follows:

(2) $${\left(\frac{d\bar{x_{N}^{b}}\left(t\right)}{\mathrm{dt}}\right)}_{i}=A_{i}P\left(t\right)k_{0}\exp\left(\frac{-E_{a}}{\mathrm{RT}}\right),$$

where: $\bar{x_{N}^{b}}\left(t\right)$—averaged nitrogen concentration in the volume of nanocrystalline, t—time, k_0_—pre-exponential coefficient, E_a_—activation energy of chemical reaction, R—gas constant, and T—temperature.

The rate of the chemical reaction in ammonia–nanocrystalline α-iron system can be described by a general equation that takes into account the influence of three parameters on the course of the chemical reaction: temperature, chemical potential of the gas phase, and nanocrystallite specific active surface area distribution:

(3) $${\left(\frac{d\bar{x_{N}^{b}}\left(t\right)}{\mathrm{dt}}\right)}_{i}=\frac{\partial \bar{x_{N}^{b}}\left(t\right)}{\partial T}\frac{\mathrm{dT}}{\mathrm{dt}}+\frac{\partial \bar{x_{N}^{b}}\left(t\right)}{\partial P}\frac{\mathrm{dP}}{\mathrm{dt}}+\frac{\partial \bar{x_{N}^{b}}\left(t\right)}{\partial A_{i}}\frac{{\mathrm{dA}}_{i}}{\mathrm{dt}}.$$

Diffusion coefficient, D, does not depend on the concentration of absorbate, and its value can be expressed by the relationship:

D = D_0_ exp(−E_a_^diff^/RT), (4)

where: D_0_—pre-exponential coefficient, and E_a_^diff^—activation energy of diffusion process.

From Equations (2) and (3), after taking into account Equation (4), the relationship follows:

(5) $${\left(\frac{d\bar{x^{b}}\left(t\right)}{\mathrm{dt}}\right)}_{i}=A_{i}D_{0}\exp\left(-\frac{E_{a}^{\mathrm{diff}}}{\mathrm{RT}}\right)\frac{E_{a}^{\mathrm{diff}}}{{\mathrm{RT}}^{2}}P\frac{\mathrm{dT}}{\mathrm{dt}}+A_{i}\frac{{\mathrm{dP}}_{\max}}{\mathrm{dt}}+D_{0}\exp\left(-\frac{E_{a}^{\mathrm{diff}}}{\mathrm{RT}}\right)P\frac{\mathrm{dA}}{\mathrm{dt}}\;.$$

The average concentration of the solute in the entire volume of a single nanocrystallite during the reaction with the gas phase can be determined using the following equation (in initial conditions, the concentration in nanocrystallite volume is 0, and the concentration on its surface is a function of time) [48]:

(6) $${\left(\frac{\bar{x^{b}}\left(t\right)}{A_{i}}\right)}_{i}=1-\frac{3D}{\frac{v}{V_{r}}r_{\max}^{2}}\exp\left(-\frac{v}{V_{r}}t\right)\left[1-{\left(\frac{\frac{v}{V_{r}}r_{\max}^{2}}{D}\right)}^{1/2}\cot{\left(\frac{\frac{v}{V_{r}}r_{\max}^{2}}{D}\right)}^{1/2}\right]+\frac{6\frac{v}{V_{r}}r_{\max}^{2}}{\pi^{2}D}\sum_{n=1}^{n}\frac{\exp\left({-\mathrm{Dn}}^{2}\pi^{2}{t/r}_{\max}^{2}\right)}{n^{2}\left(n^{2}\pi^{2}-\frac{v}{V_{r}}r_{\max}^{2}/D\right)},$$

assuming that the adsorbate concentration in the gas phase, x_N_^g^(t), varies according to the leaching model with perfect mixing:

(7) $$P\left(t\right)=x_{N}^{g}\left(t\right){=x}_{N,0}^{g}\left[1-\exp\left(-\frac{v}{V_{r}}t\right)\right],$$

where: v—gas flow rate, V_r_—reactor volume, $x_{N,0}^{g}$—maximum concentration in the gas phase, and adsorbate surface concentration changes over time according to:

ln x_N_^s^ = K_ad_ lnP ≈ ln A. (8)

**Figure 3:**
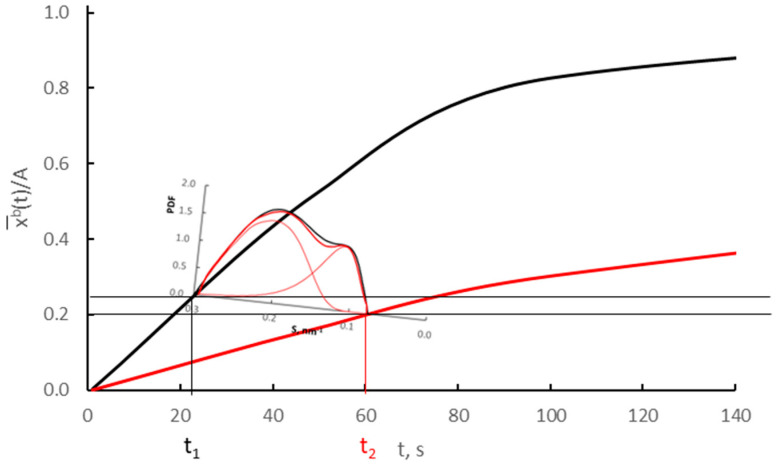
Change in the average concentration of nitrogen in the volume of iron nanocrystallites with an active specific surface area 0.15 nm^−1^ (black color) and 0.04 nm^−1^ (red color) as $\bar{x^{b}}\left(t\right)/A$ = f(t) with a diffusion coefficient D = 0.1 nm^2^/s.

Calculation results derived from Equation (6), for two exemplary iron nanocrystallites of 10 nm (A_i_ = 0.15 nm^−1^) and 40 nm in radius (A_i_ = 0.04 nm^−1^), with value of the parameter v/V_r_ = 0.03 s, are shown in Figure 3 as the change of $\bar{x^{b}}\left(t\right)/A$ depending on time. For modeling, the value of coefficient of diffusion by the boundary layer of surface iron atoms of D = 0.1 nm^2^/s was assumed so that the reaction rates during the experiment and in modeling were the same. Figure 3 also shows the active specific surface area distribution density probability in the catalyst heated at 500 °C.

**Figure 4:**
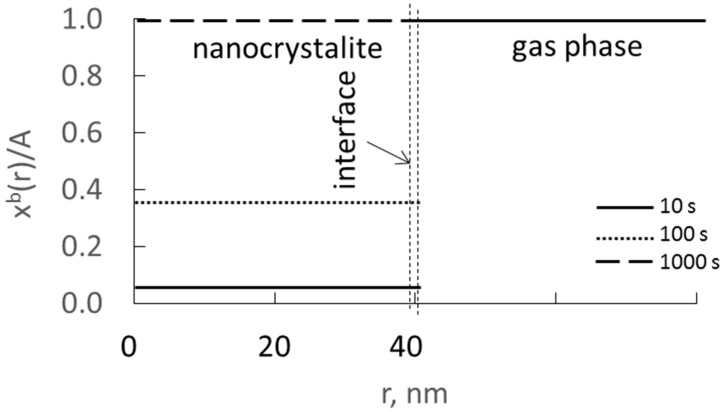
Dependence of nitrogen concentration in nanocrystallite volume (radius 40 nm) on radius and time at a constant surface concentration and with a diffusion coefficient of D = 0.1 nm^2^/s.

It can be assumed that, under the conditions of the nitriding process, when nitriding potential P(t) >> P_0_, on the surface of iron nanocrystallites, the nitrogen concentration is maximum and constant until the phase transformation of α-Fe(N) to nitride γ’-Fe_4_N. Surface concentration x_N_^s^ will be constant and greater than the equilibrium concentration determined by the equilibrium between the surface and the volume of the nanocrystallite. Process rate is limited by diffusion through the boundary layer of surface iron atoms, and, in volume of iron nanocrystallite, a constant level of nitrogen concentration x_N_^b^ is quickly established at a constant maximum during the experiment gas phase nitriding potential. The dependence of the nitrogen concentration in nanocrystallite volume on the process time at a constant surface concentration of adsorbate, x_N_^s^, is presented schematically in Figure 4.

The rate at which iron nanocrystallite saturates with nitrogen depends on the difference in surface concentration and the concentration in volume of the nanocrystallite according to Fick’s equation:

(9) $${\left(\frac{d\left(\frac{\bar{x_{N}^{b}}\left(t\right)}{A_{i}}\right)}{\mathrm{dt}}\right)}_{i}=D\left(x_{N,i}^{s}-\bar{x_{N,i}^{b}}\left(t\right)\right)=D\left(A_{i}-\bar{x_{N,i}^{b}}\left(t\right)\right).$$

The total concentration of the absorbate in the volume of sample of nanocrystallites, x^b^, for the dependence on temperature, chemical potential of the gas phase, and crystallite size described by continuous functions is as follows:

(10) $$\bar{x_{N,i}^{b}}\left(t\right)=\int_{T0}^{T}\int_{P0}^{P}\int_{A0}^{A}{\left(\frac{d\bar{x_{N}^{b}}\left(t\right)}{\mathrm{dt}}\right)}_{i}{\mathrm{dTdPdA}}_{i}.$$

In the range of one α phase, at constant temperature and at constant potential of the gas phase, the averaged reaction rate can be written as:

(11) $${\left(\frac{\Delta \bar{x_{N}^{b}}\left(t\right)}{\Delta t}\right)}_{i}=n_{N,i}^{b}N_{i}\left(\frac{\int_{x_{N}^{b}=0}^{x_{N}^{b}}{\left(\frac{d\bar{x_{N}^{b}}\left(t\right)}{\mathrm{dt}}\right)}_{i}^{}}{n_{\mathrm{Fe},i}^{b}{\Delta t}_{i}}+C_{1}+C_{2}\right),$$

where: n—number of moles, N_i_—number of nanocrystallites in the i-th fraction of nanocrystallites in the sample, and C_1_, C_2_—constants taking into account the influence of temperature and gas phase potential on the reaction rate.

After substituting Equations (9)–(11), the average rate can be calculated as:

(12) $${\left(\frac{\Delta \bar{x_{N}^{b}}\left(t\right)}{\Delta t}\right)}_{i}=n_{N,i}^{b}N_{i}\left(\frac{D\int_{x_{N}^{b}=0}^{x_{N}^{b}}{\left(x_{N}^{s}-\bar{x_{N}^{b}}\left(t\right)\right)}_{i}^{}\mathrm{dt}}{n_{\mathrm{Fe},i}^{b}{\Delta t}_{i}}\right)=n_{N,i}^{b}N_{i}\left(\frac{D\int_{x_{N}^{b}=0}^{x_{N}^{b}}{\left(A-\bar{x_{N}^{b}}\left(t\right)\right)}_{i}^{}\mathrm{dt}}{n_{\mathrm{Fe},i}^{b}{\Delta t}_{i}}\right).$$

The concentration in volume of nanocrystallite during the process will change according to the following equation:

(13) $$\bar{x_{N}^{b}}\left(t\right)=x_{N}^{s}-\exp\left(-\mathrm{Dt}\right).$$

**Figure 5:**
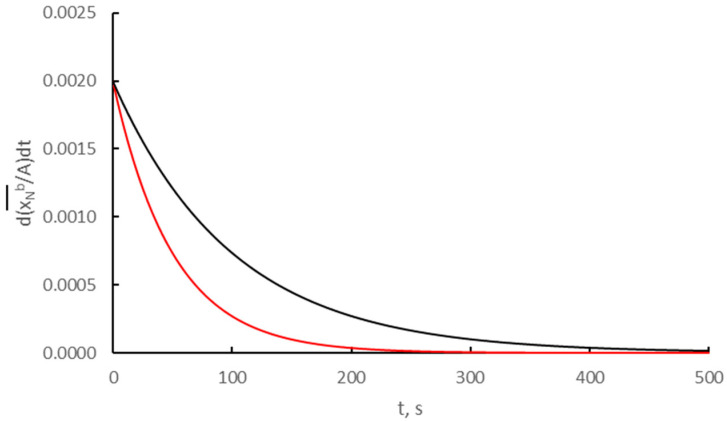
Process rates on two different crystallites with a specific active surface area 0.15 nm^−1^ (black color) and 0.04 nm^−1^ (red color) as $d\left(\frac{\bar{x^{b}}\left(t\right)}{A}\right)/\mathrm{dt}$ = f(t) with a diffusion coefficient D = 0.1 nm^2^/s.

In the results obtained for two crystallites, the average rate of the absorption process (Figure 5) was determined according to Equation (12).

**Figure 6:**
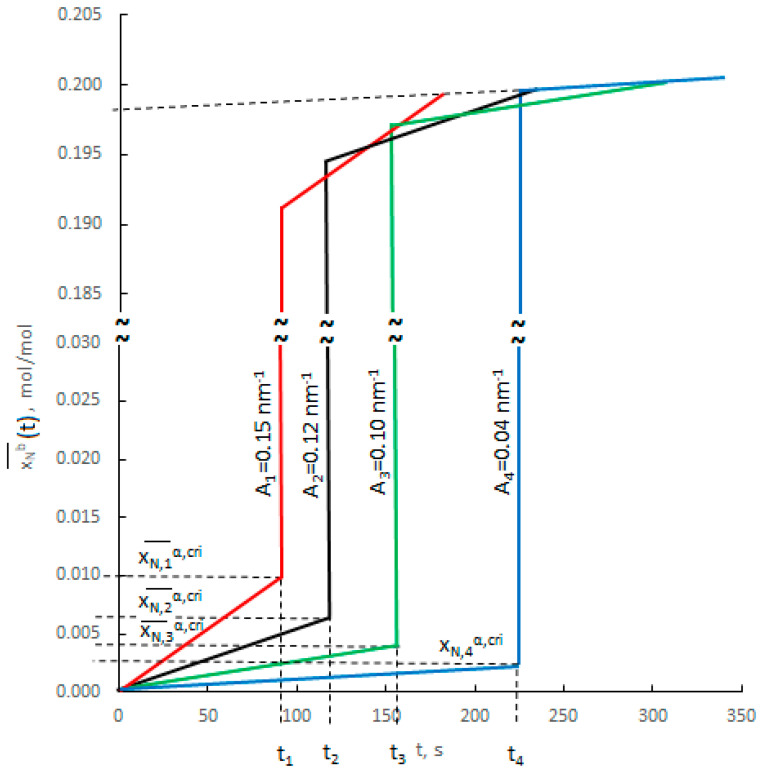
Changes in nitrogen concentration over time for nanocrystallites with active specific surfaces in the range 0.04–0.15 nm^−1^.

Figure 6 shows, schematically, changes in nitrogen concentration over time for nanocrystallites with an active specific surface in the range of 0.04–0.15 nm^−1^.

In the kinetic region of chemical reaction, the critical concentration in volume of a nanocrystallite with a larger active specific surface area will be achieved faster than in the nanocrystallite with a smaller active specific surface, although, according to the results of thermodynamic studies [44,45], the critical concentration for the latter nanocrystallite is lower than for the first.

The measured reaction rate is the sum of the process rates on individual i-th nanocrystallites in the sample:

(14) $$\frac{d\bar{x^{b}}\left(t\right)}{\mathrm{dt}}=\sum_{i=\min}^{i=\max}n_{i}^{b}N_{i}{\left[\frac{d\bar{x^{b}}\left(t\right)}{\mathrm{dt}}\right]}_{i}=\sum_{i=\min}^{i=\max}n_{i}^{b}N_{i}{\left[\frac{{\mathrm{dn}}^{b}\left(t\right)}{n_{i}^{b,\alpha }\mathrm{dt}}\right]}_{i}=\sum_{i=\min}^{i=\max}f\left(\mathrm{nASD}\right){\left[\frac{{\mathrm{dn}}^{b}\left(t\right)}{\mathrm{dt}}\right]}_{i}.$$

The actual system consists of a set of crystallites described by the distribution density probability of their sizes characterized by the specific active surface area of the crystallite, A. It was assumed in the paper that this is a bimodal distribution consistent with experimental tests [33,38,47,49] and model calculations [44,45] for the nanocrystalline iron-ammonia-hydrogen system.

In the nitriding process, catalytic decomposition of ammonia takes place parallel to nitriding reaction. At high process temperatures, the surface diffusion rate is so high that, in stationary states, the chemical composition of the surface does not depend on the size of iron nanocrystallites.

In the stationary states (the composition of the gas and solid phases does not change with time, and the degree of nanocrystalline substance conversion is a function of temperature and chemical potential of the gas phase), the rate of ammonia decomposition depends on the temperature, nitriding potential of the gas phase, and degree of nitriding of the nanocrystalline iron sample. On the mixture of phases α-Fe(N) and nitride γ’-Fe_4_N, the reaction rate of the catalytic decomposition of ammonia is described by an empirical linear equation [11,50]:

r_d_ = 6.2·10^−6^ − 2.3·10^−6^ lnP = k K_d_ S_a_ (ϑ – lnP), (15)

which, assuming ϑ → 0 at P → 0, can be transformed into the form:

r_d_ = −k K_d_ A V lnP = (k K_d_/R T) A V μ_N_^g^, (16)

where: k—empirical reaction rate constant of catalytic decomposition of ammonia, K_d_—equilibrium constant of the catalytic decomposition of ammonia, S_a_—total active surface of all nanocrystallites, A—total active specific surface of all nanocrystallites, ϑ—maximum active surface area at P → 0, and μ_N_^g^—gas phase nitogen potential.

In order to determine the real, active specific surfaces (which depends on the conditions of the recrystallization process), the degree of conversion in the catalytic decomposition of ammonia, α_NH3_, was calculated:

(17) $$\alpha_{\mathrm{NH}3}=\frac{F_{0H2}-x_{H2}F_{0}}{F_{0\mathrm{NH}3}{\;x}_{H2}-1{.5\;F}_{0\mathrm{NH}3}},$$

where: F_0H2_, F_0NH3_, F_0_—flow rates of hydrogen, ammonia, and summary gas stream, respectively, at the reactor inlet, and x_H2_—hydrogen concentration in gas phase.

Mole fraction of ammonia in the reaction mixture, x_NH3_,

(18) $$x_{\mathrm{NH}3}=\frac{F_{\mathrm{NH}3}^{0}-\alpha_{\mathrm{NH}3}F_{0\mathrm{NH}3}^{}}{F^{0}+\alpha_{\mathrm{NH}3}F_{0\mathrm{NH}3}^{}},$$

was determined on the basis of the mass balance of the reactor in stationary states [50,51].

The rate of catalytic decomposition of ammonia, taking into account the degree of ammonia conversion and the ammonia flow rate at the inlet to the reactor, F_0NH3_, was calculated according to the following formula [50,51]:

(19) $$r_{d}=\alpha_{\mathrm{NH}3}F_{0\mathrm{NH}3}^{}.$$

**Figure 7:**
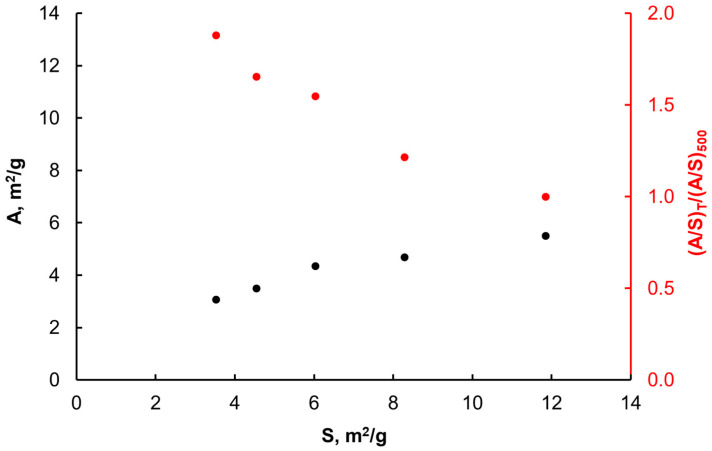
Values of the active specific areas A calculated with the use of measurements of the catalytic decomposition rates of ammonia and ratios of (A/S)_T_/(A/S)_500_ for samples reduced and annealed in hydrogen at various temperatures in the range 500–700 °C as a function of the specific surface area S.

After comparing Equations (16) and (19) and using experimental data on active sites concentration [11,52], the total active specific surface area of the samples of the iron catalyst reduced and heated in hydrogen at different temperatures was determined, taking into account the rate of catalytic decomposition of ammonia (Figure 7). Based on the ratio of the active iron surface to the specific iron surface, the degree of coverage of the catalyst surface with promoters was determined.

Morphology of nanocrystalline iron in the catalyst changes with the increase in the temperature of reduction and annealing of the samples in hydrogen: both the specific and active surface area decrease, and the proportion of the surface not taken up by the promoters increases.

Nanocrystallites obtained at constant temperature and in different promoter potentials P_PR,1_–P_PR,2_ are characterized by different specific surfaces, according to the conditions of their synthesis (temperature and promoter potential), and may undergo a structure change in result of recrystallization when the potential is reduced to P_PR,3_ due to decreasing the potential or increasing the temperature. Mass of the recrystallizing nanocrystallites with the greatest specific active surface, unstable at lower potentials, remains in the system. However, the new active surface distribution will be narrower with a smaller mean value. The total area of all nanocrystallites in the system, S_t_, related to 1 mol of α-Fe phase will be determined by the cumulative distribution function (CDF) assigned to the probability distribution characteristic of the nanocrystalline system under the given conditions of potential P and temperature:

(20) $$\mathrm{CDF}=\int_{A_{\min}}^{A_{\max}}\frac{1}{\sigma \sqrt{2}\pi }\exp\left(\frac{-{\left(A-A_{m}\right)}^{2}}{{2\sigma }^{2}}\right)\mathrm{dA}=\frac{1}{2}\left(1+\mathrm{erf}\frac{\left(A_{\max}-A_{\min}\right)-A_{m}}{\sigma \sqrt{2}}\right),$$

where: A_min_—specific active surface corresponding to minimum potential in the system, A_max_—specific active surface corresponding to maximum potential in the system, and erf—Gauss error function.

**Figure 8:**
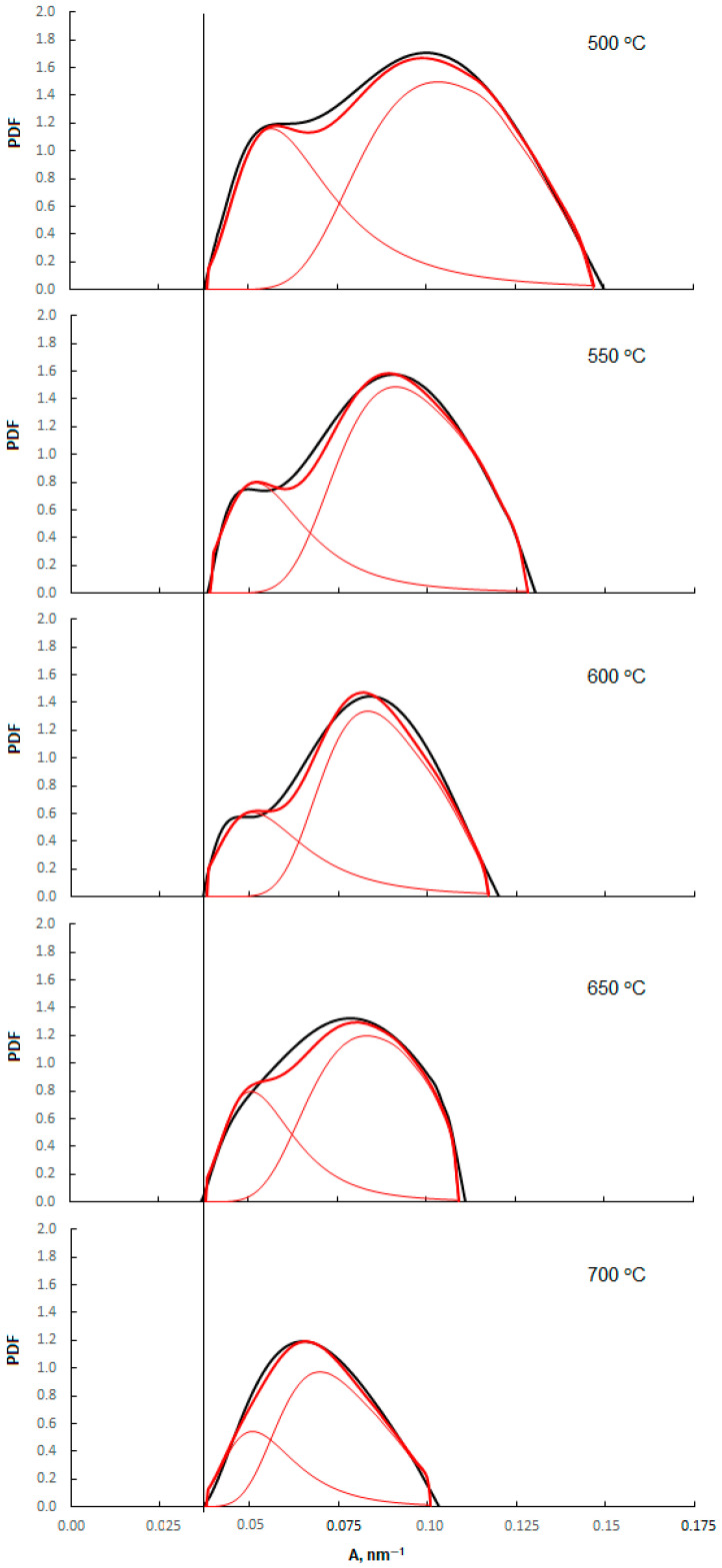
Distribution density probability of active specific surfaces of nanocrystalline iron samples annealed at temperatures in the range of 500–700 °C.

Based on the measurements of the nitriding reaction rate of nanocrystalline iron heated in hydrogen at various temperatures in the range 500–700 °C, the distribution density probabilities of iron nanocrystallites with certain active specific surfaces in the tested samples were determined (Figure 8)—Equations (9) and (11). The results of measurements of the specific surface of samples annealed at different temperatures and the parameters of model distribution density probabilities of nanocrystallites are presented in Table 1.

As a result of overheating, the smallest nanocrystallites transform into larger, stable ones, whose active specific surface A is determined by the equilibrium established under given conditions; therefore, the limit values of nanocrystallites with the largest active specific surfaces A^max^ change in the range 0.15–0.10 nm^−1^. The size of larger than minimal nanocrystallites remaining in the sample is not the result of the equilibrium state; only the equilibrium between the promoters and the nanocrystallite surface will be established there, but the S/V relationship of the nanocrystallites will not meet the equilibrium conditions [53]. The largest nanocrystallite in the catalyst does not change as a result of the overheating process, and its active specific surface area A^min^ in each sample is 0.04 nm^−1^.

## 5. Conclusions

Based on the results of measurements of the nitriding rate obtained for the samples after the overheating in hydrogen in the temperature range of 500–700 °C, the distributions density probabilities of the active specific surfaces of iron nanocrystallites of the tested samples were determined. The determined distributions are bimodal and are described by the sum of two Gaussian distribution functions, wherein the largest nanocrystallite in the overheating process does not change, and the size of the smallest nanocrystallites increases with the increase of the recrystallization temperature.

Parallel to the nitriding reaction, catalytic decomposition of ammonia takes place in direct proportion to the active surface of the iron nanocrystallite. Based on the ratio of the active iron surface to the specific iron surface, the degree of coverage of the catalyst surface with promoters was determined.

## Figures and Tables

**Table 1:** Specific surfaces and parameters of two model Gaussian distributions that make up the total distribution characterizing the samples reduced and heated in hydrogen at various temperatures in the range of 500–700 °C.

Heating Temperature °C	Specific Surface Area m^2^ g^−1^	The Time to Start the Transformation α→γ’ s		Nanocrystallite A Distribution Parameters					
				Peak No. 1			Peak No. 2		
		For the Nanocrystallite with the Largest A	For the Nanocrystallite with the Smallest A	Sigma nm	DN nm	A^max^ nm^−1^	Sigma nm	DN nm	A^min^ nm^−1^
500	12	90	230	8	29	0.15	12	58	0.04
550	9	85		7.5	33	0.13			
600	7	75		7	36	0.12			
650	6	80		9	36	0.11			
700	4	70		9	43	0.10			
